# Covidalism^©®^: COVID Restrictions in USA have no Health Benefits at all

**Published:** 2024-11-08

**Authors:** Jan Charles Biro

**Affiliations:** Homulus Foundation, Los Angeles, United States

**Keywords:** COVID-19, Pandemic, Political, Mortality, Age, Aging, Co-morbidity, Underlying cause of death, UCOD, USA, States, Restrictions, Political score, Restriction ranking, Religion, ethnicity

## Abstract

**Background:**

Shortly after the detection of the COVID virus in January 2020 the US Government introduced and enforced a series of restrictions to protect the elderly from a “deadly virus” and the “pandemic of the century”. Persons who disagreed were silenced and punished.

**Objective:**

In the 15th month of COVID epidemic (April 2021), we have access to sufficient statistical data and methods to better understand the nature, origin of the COVID pandemic. It is now possible to reliably evaluate the effectiveness of the restrictions and the human factors/driving forces behind this drastic limitation of our natural freedom in this country.

**Methods:**

Publicly available epidemiological and population parameters were collected and analyzed using the ‘political score’ and ‘restriction ranking’ of 50 states and evaluated by simple and usual statistical methods, mainly correlation analyses.

1. The Political Score of the States (D/R) is the ratio of the number of citizens who self-identified as democrats (D) or republicans (R). The political scores of the 50 states altogether defined a wide, continuous scale, the political scale, that could be used to measure (and statistically evaluate) the effect of politics of a state on the numerical parameters of that state, including population and epidemiologic parameters.
2. The COVID Restriction Score of the States were created to rank the states from 1-50, there 1 is the lowest number of restrictions and 50 is the highest number of restrictions applied by the States. It was based on 13 different key metrics.

**Results:**

This study revealed, that 1) restrictions reduced the number of viral infections, but 2) they totally failed to reduce the number of supposedly COVID related deaths, expressed as mortality, 3) they increased (SIC!) the lethality of coronavirus. The grade of restrictions were/are strongly associated to the 4) left/right political ratio of the States, there States with more democrat citizens practiced more restrictions. It was found that race, religion and Medicare/healthcare spending have significant influence on politic as well as on the grade of restriction orders. Factors moving States toward the political left and harder COVID restrictions have 5) larger ‘non-white’ population, 6) larger number of ‘non-protestant’ believers, 7) larger Jewish population, and 8) more generous Medicare/healthcare spending. It was not possible to see any influence of the size of the 9) senior (65+) population [i.e. those who are allegedly the most vulnerable and are mostly in need of protective restrictions] neither on the States politic nor on the restrictions.

**Conclusions:**

The degree of restrictions enforced by different states ware not primarily determined by biological or epidemiological factors (like number of elderly, say 65+ in the state) but by social, political influences instead. Political (a), religious (b) ethnic (c) and economic (d) forces represented the decisive forces on the state’s restrictive orders and not the convincing evidence of the potentially harmful effects of the COVID infection and the well-founded adequacy of the defense against it.

## Introduction

Today, in the 15th month of COVID epidemic, we have now access to sufficient statistical data and methods to better understand the nature, origin and driving forces behind this pestilence. Introduction of ‘political score’ and ‘restriction ranking’ of 50 states and comparing the COVID data and the result of restrictions under different circumstances are very valuable sources of reliable information to see through the massive fog created by the occasionally dishonest media, sometimes fake scientists and the usually ignorant politicians. This study revealed, that 1) restrictions reduced the number of viral infections, but 2) they totally failed to reduce the number of supposedly COVID related deaths, expressed as mortality, 3) they increased (SIC!) the lethality of coronavirus. The grade of restrictions were/are strongly associated to the 4) left/right political ratio of the States, there States with more democrat citizens practiced more restrictions. It was found that race, religion and Medicare spending have significant influence on politic as well as on the grade of restriction orders. Factors moving States toward the political left and harder COVID restrictions have 5) larger ‘non-white’ population, 6) larger number of ‘non-protestant’ believers, 7) larger Jewish population, and 8) more generous Medicare spending. It was not possible to see any influence of the size of the 9) senior (65+) population [i.e. those who are allegedly the most vulnerable and are mostly in need of protective restrictions] neither on the States politic nor on the restrictions. The conclusion is that COVID restrictions are primarily politically motivated, they are ineffective to reduce COVID related mortality and they provide no extra health benefit for the so called “vulnerable” minority.

## Materials and Methods

COVID statistical data was collected from public sources, like Center for Disease Control, CDC and Worldometer [1,2]. The Political Score of the States (D/R) is the ratio of the proportion of citizens who self-identified as democrats or lean toward democratic party (D) divided by the proportion of citizens who self-identified as republican or lean toward republican party (R) in a given State [3]. The lowest Score (0.4) indicates a State with the lowest number of democrat oriented (D) or the highest number of republican oriented (R) citizens. The highest D/R score (max. 2.07) is obtained for a state with the highest number of D-oriented or the lowest number of R-oriented citizens. This tool turned out to be very useful to detect socio-political factors involved in the COVID syndemic [4,5]. The COVID Restriction Score of the States were adopted from a recent publication [6]. It ranks the states from 1-50, there 1 is the lowest number of restrictions and 50 is the highest number of restrictions applied by the States. The results were evaluated by standard statistical methods.

## Results

### Correlation between political and restriction scores and some epidemiologic parameters of the COVID pandemic in the states of USA

**Figure 1:**
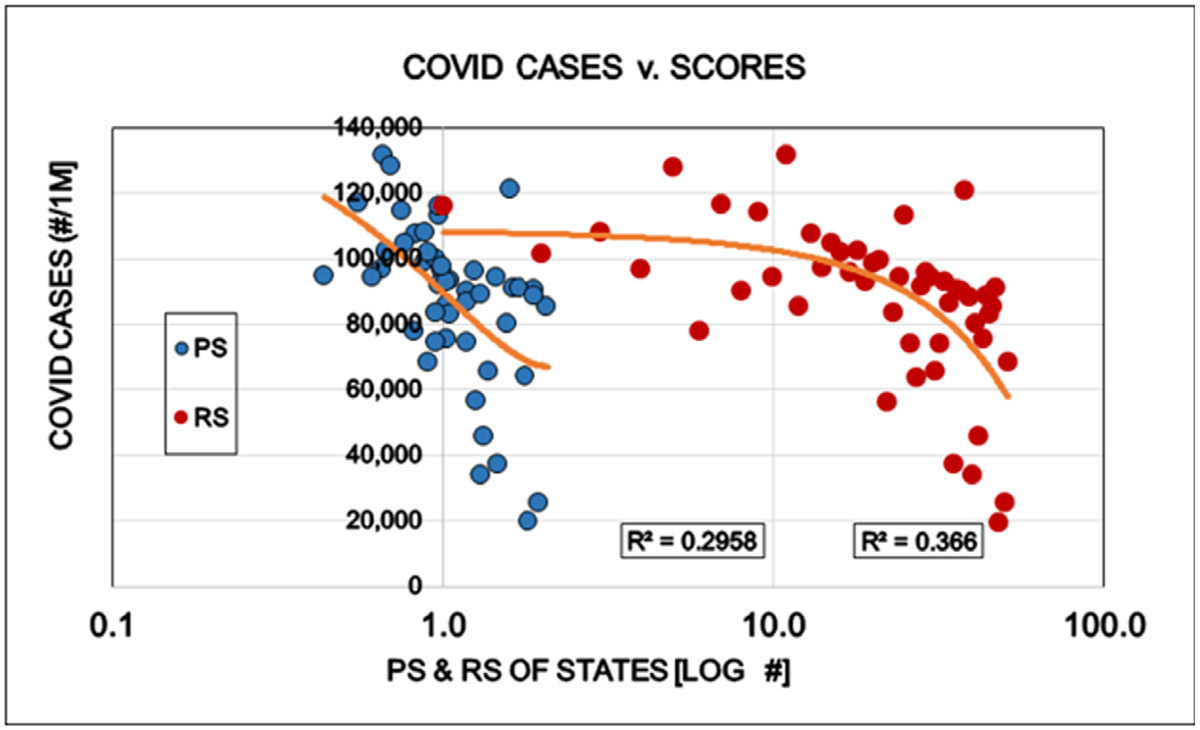
Effects of politics (Political Score, PS: Democrat/Republican Ratio) and restrictions (Restriction Score, RS) on the total number of COVID cases in the States of the USA

**Figure 2:**
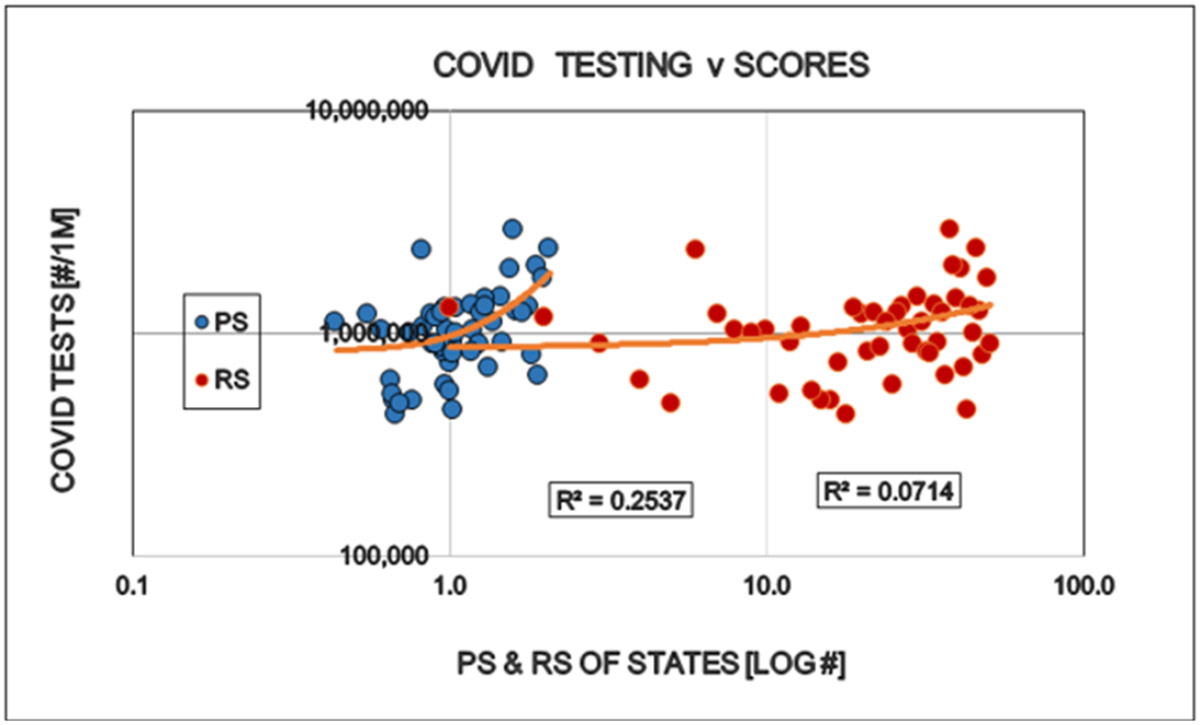
Effects of politics (Political Score, PS: Democrat/Republican Ratio) and restrictions (Restriction Score, RS) on the total number of specific laboratory COVID viral tests in the States of the USA

**Figure 3:**
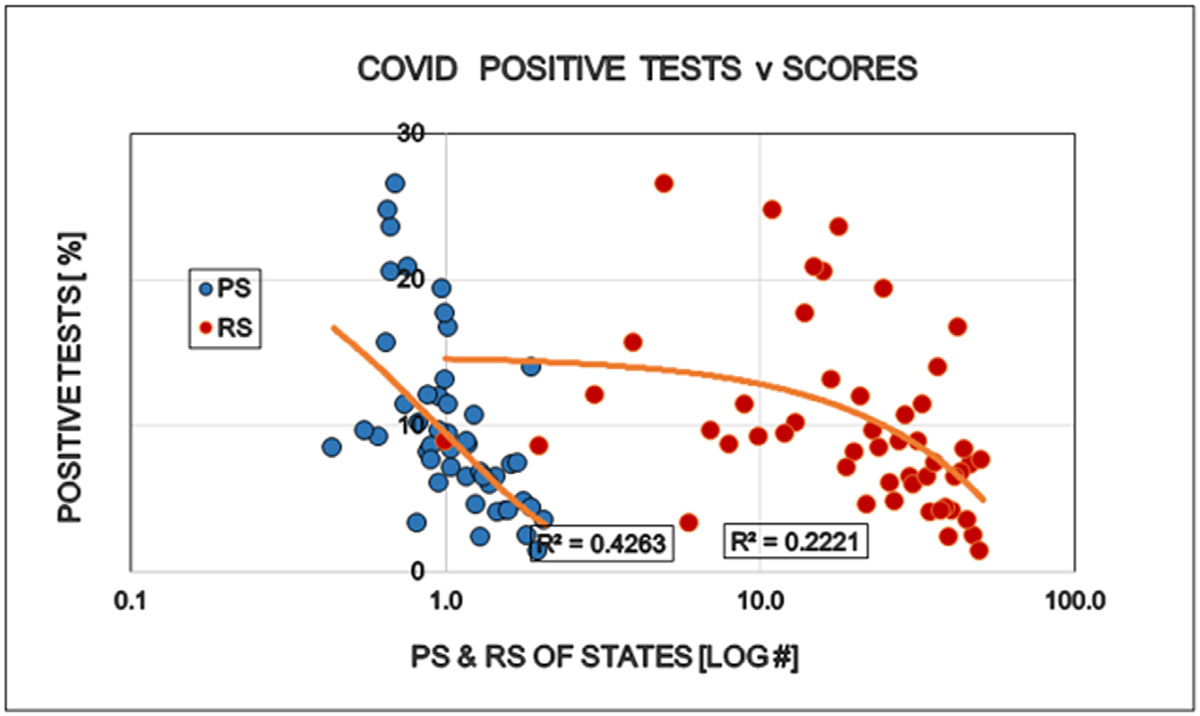
Effects of politics (Political Score, PS: Democrat/Republican Ratio) and restrictions (Restriction Score, RS) on the proportion (%) of positive total specific laboratory COVID viral tests in the States of the USA

**Figure 4:**
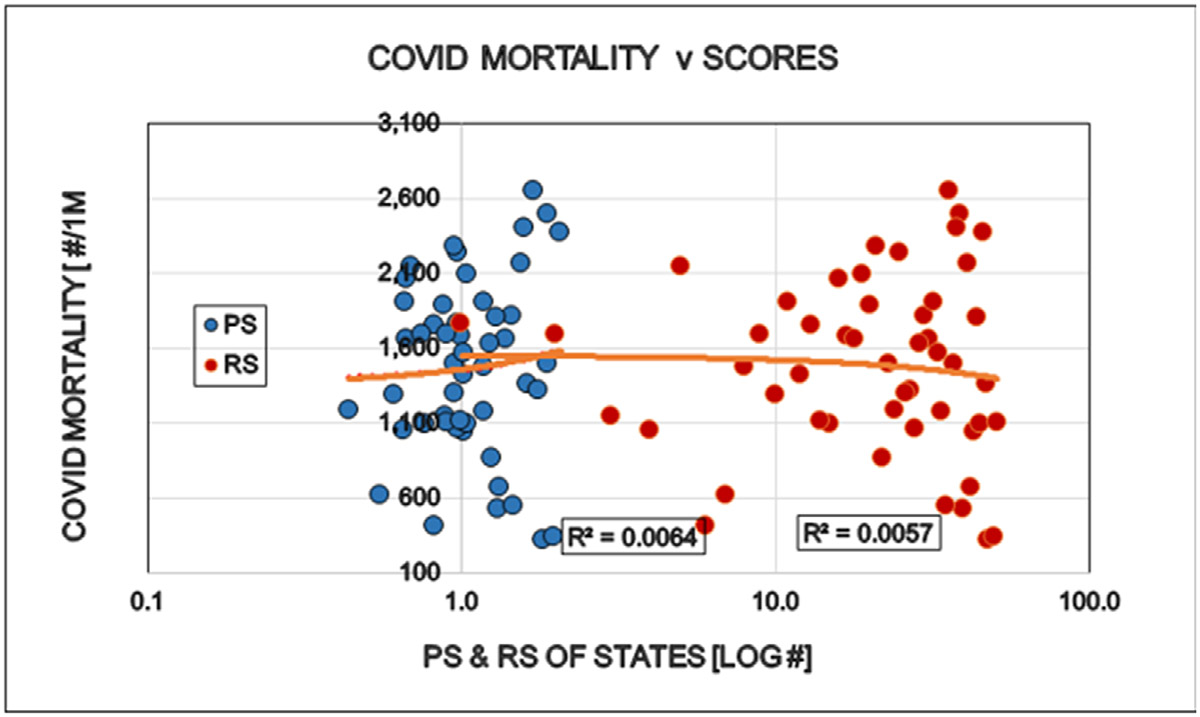
Effects of politic (Political Score, PS: Democrat/Republican Ratio) and restrictions (Restriction Score, RS) on the COVID related mortality in the States of the USA

**Figure 5:**
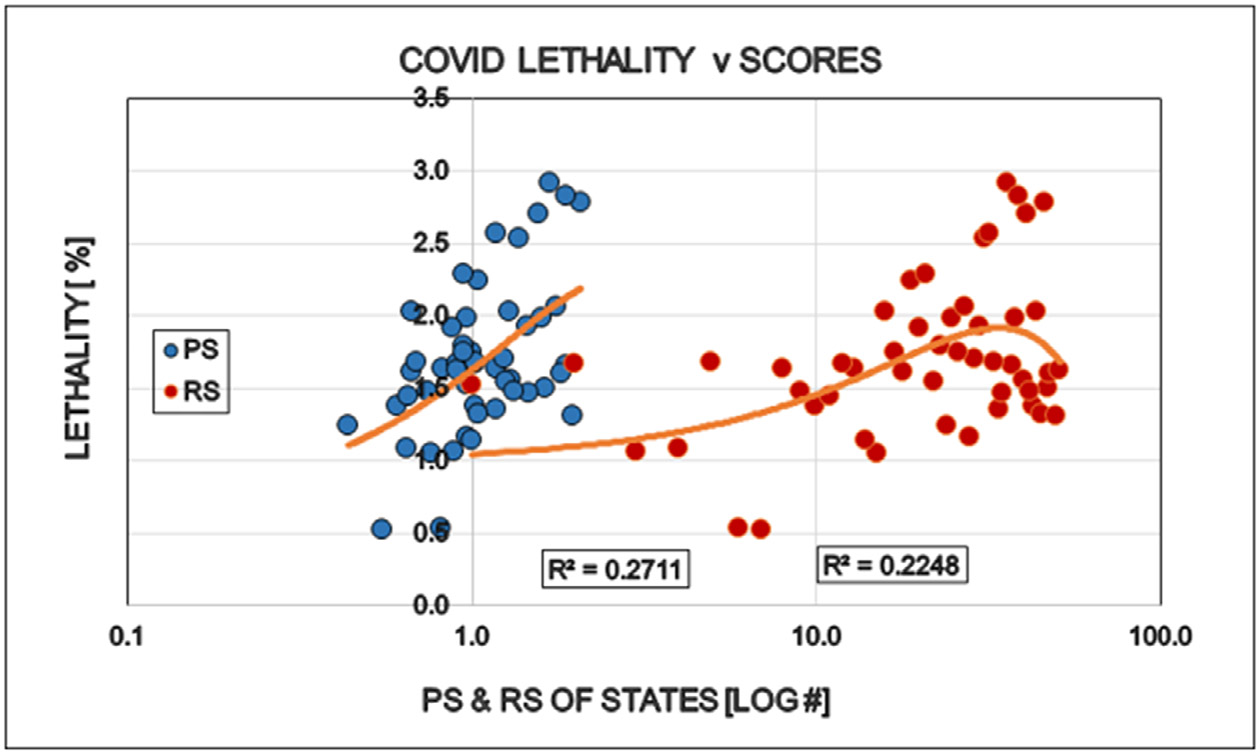
Effects of politic (Political Score, PS: Democrat/Republican Ratio) and restrictions (Restriction Score, RS) on the COVID related lethality in the States of the USA

Restrictions reduced the number of COVID cases (i.e. persons positive with viral tests). This is well in line with the purpose of the restrictions and might be interpreted as supporting argument for the established approach to the epidemic (Figure 1). The number of specific laboratory COVID viral tests was not convincingly increased by the restrictions the proportion (%) of positive viral tests decreased as expected (Figures 2 and 3). However the mortality of COVID remained largely unaffected by the restrictions. The statistical results confirm the original observation by other investigators [6,7] that restrictions are NOT able to reduce the detectable number of COVID deaths (Figure 4). A different insight emerged when the number of COVID deaths were related to the number of persons diagnosed with the virus infection, i.e. the lethality was calculated. The lethality of the virus increased (SIC!) with increasing degree of restrictions as well as higher political score. This is an intriguing observation, because it may suggest the dissociation between determining COVID as the UCOD and diagnosing the COVID infection itself (with or without fatal outcome) (Figure 5).

### Possible determinants of the state’s politic and degree of COVID restrictions

**Figure 6:**
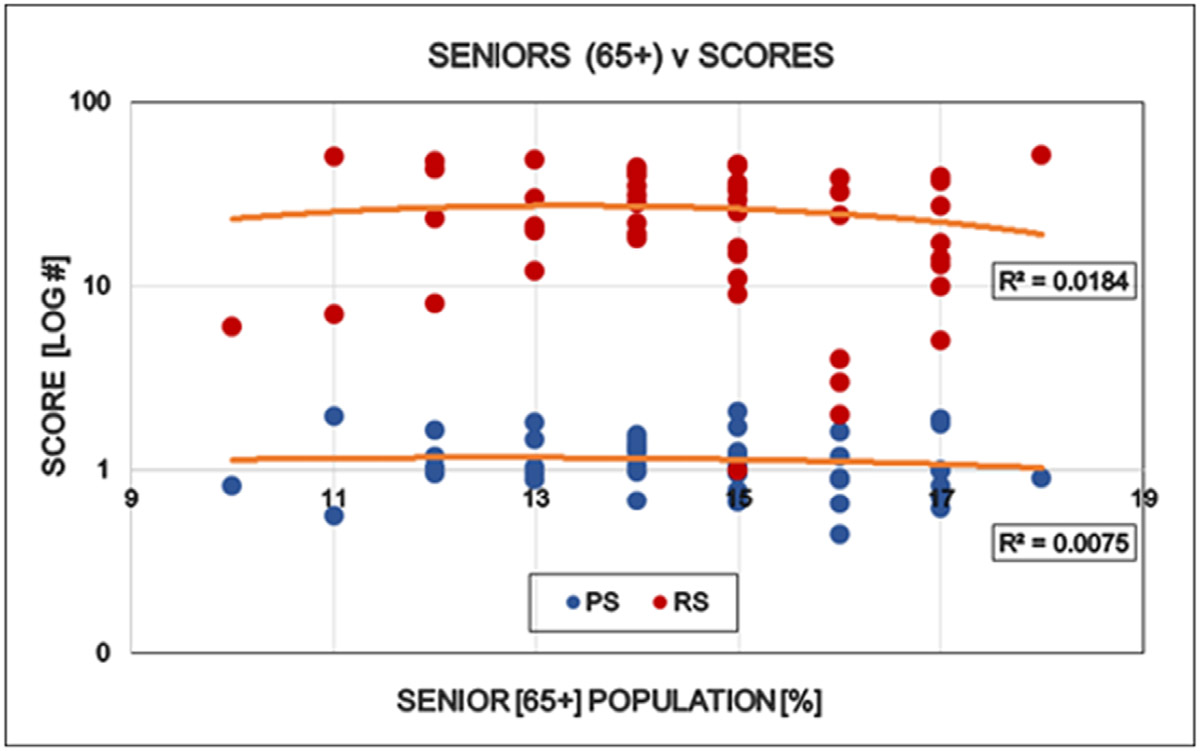
Effect of the size of the senior population (65+) in the States on the politics (Political Score, PS: Democrat/Republican Ratio) and restrictions (Restriction Score, RS) of that State

**Figure 7:**
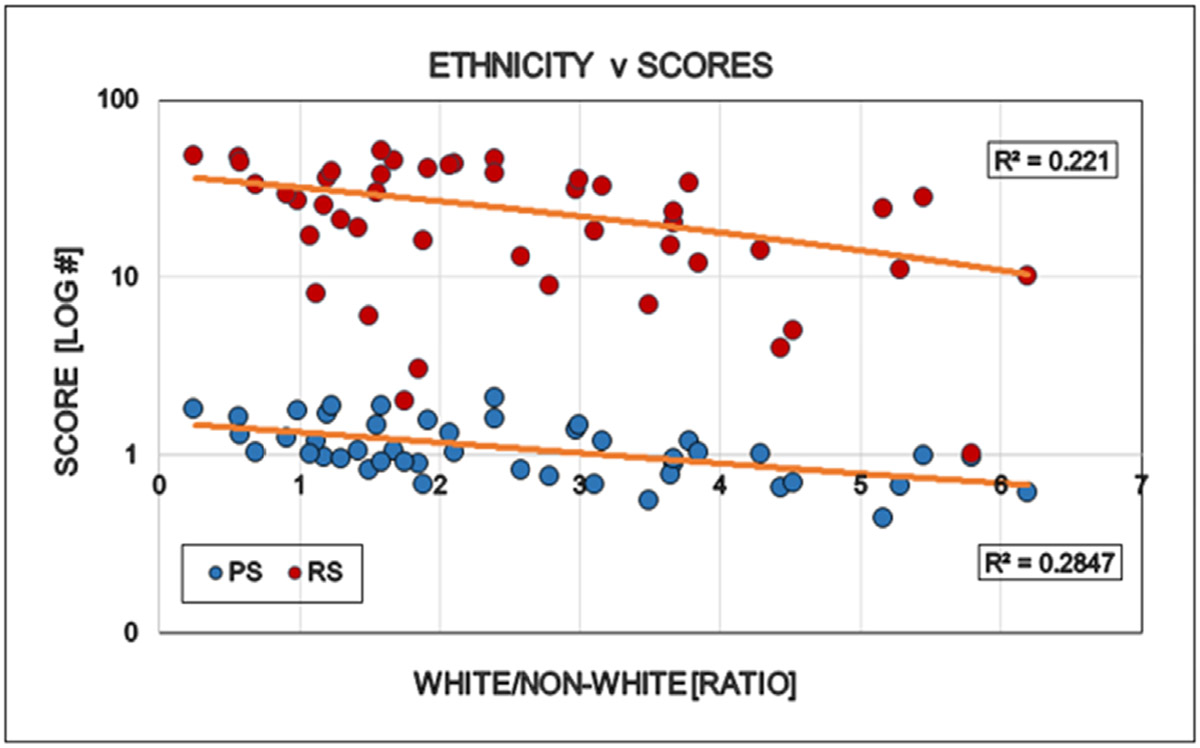
Effect of ethnicity in the States on the politics (Political Score, PS: Democrat/Republican Ratio) and restrictions (Restriction Score, RS) of that State

**Figure 8:**
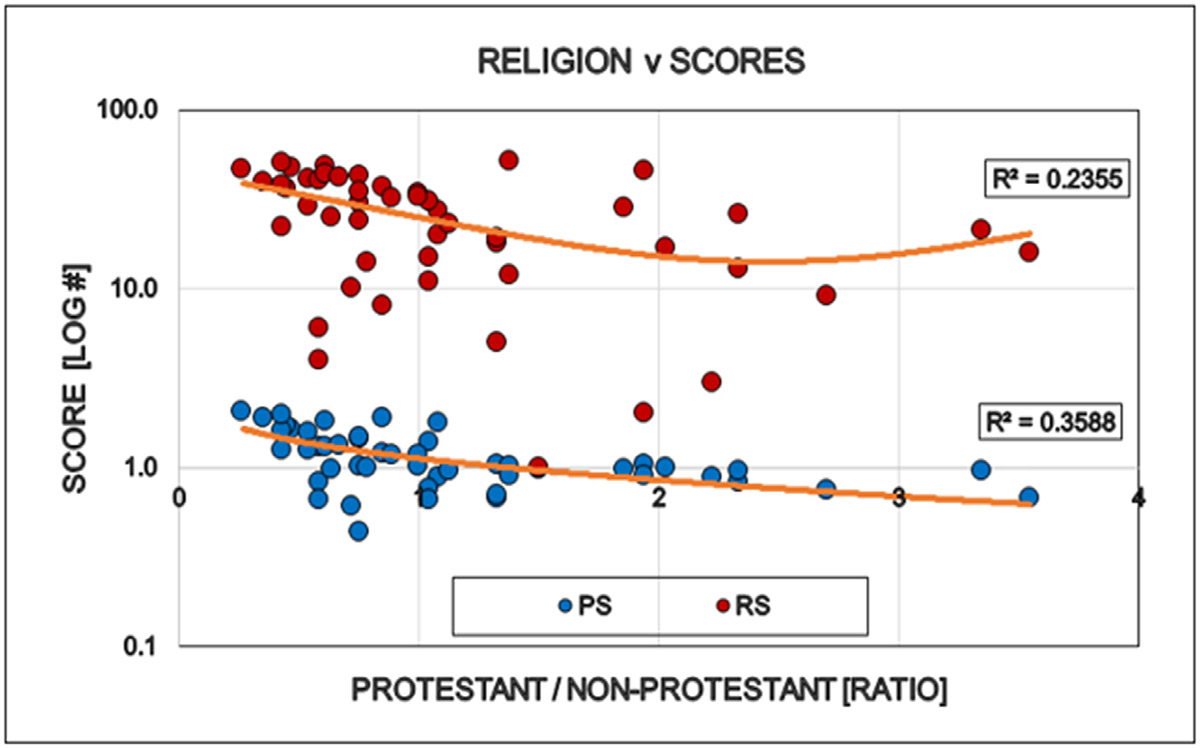
Effect of religious affiliation in the States on the politics (Political Score: Democrat/Republican Ratio) and restrictions (Restriction Rank) of that State

**Figure 9:**
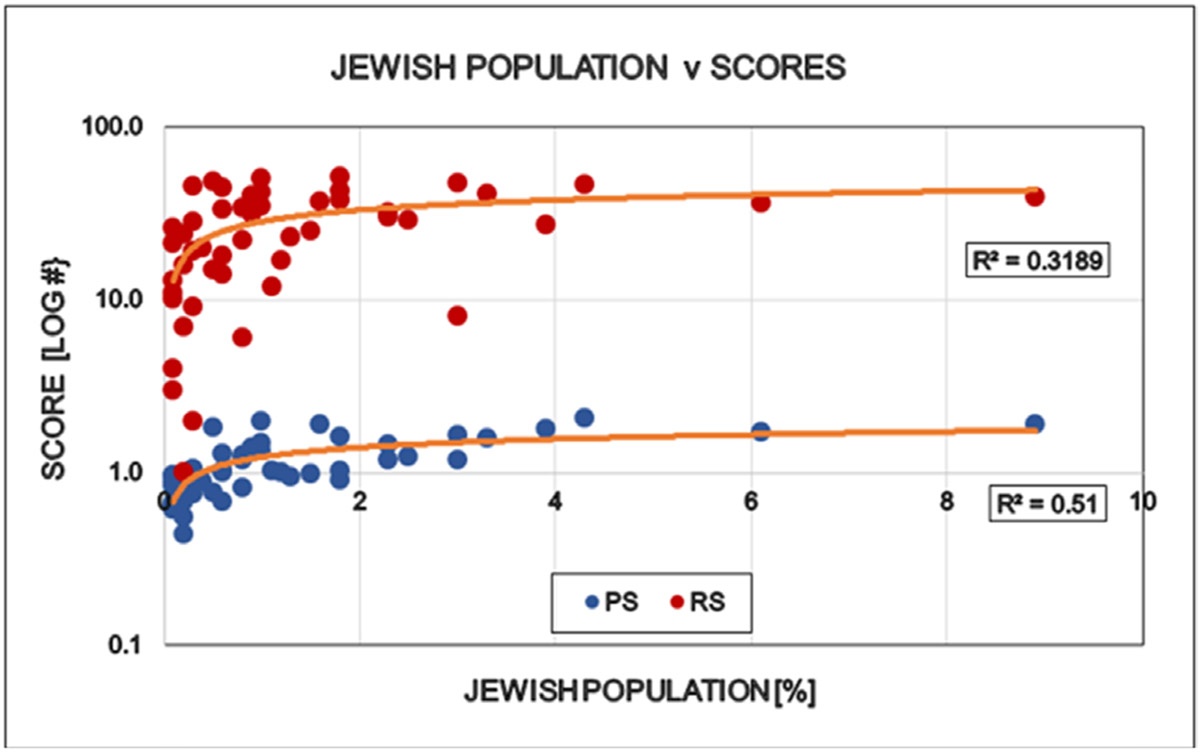
Effect of Jewish population in the States on the politics (Political Score, PS: Democrat/Republican Ratio) and restrictions (Restriction Score, RS) of that State

**Figure 10:**
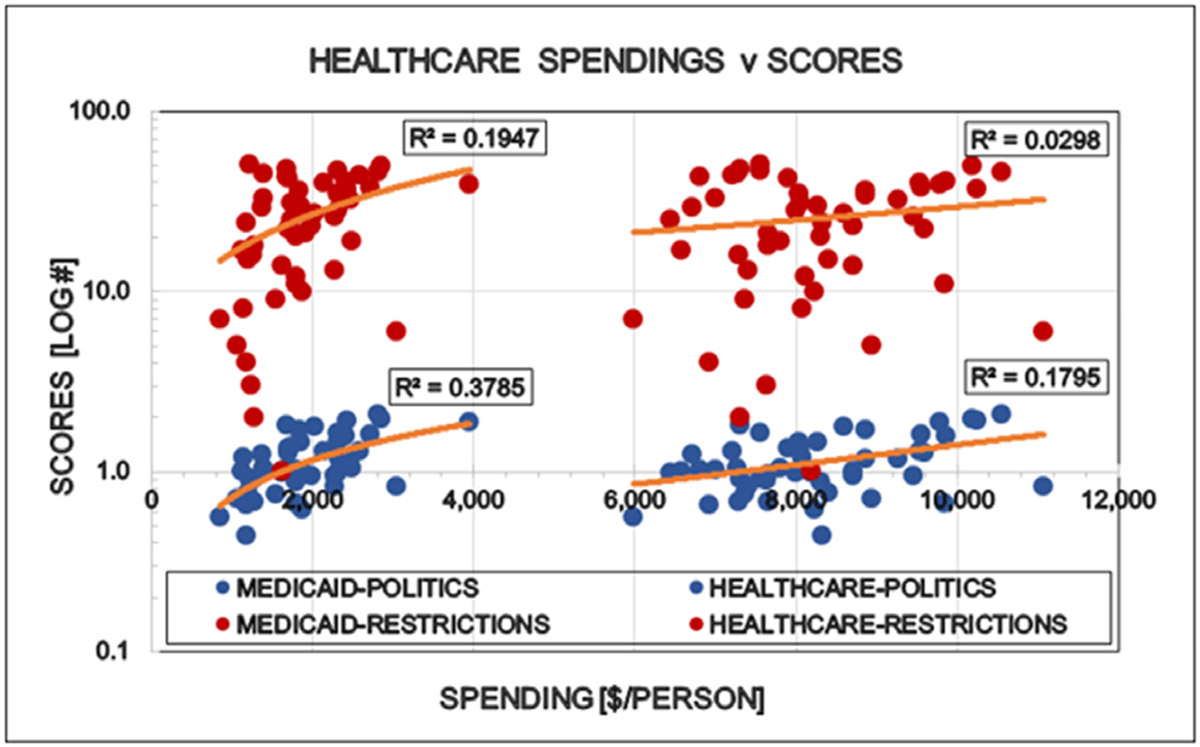
Correlation between healthcare spending (all and Medicaid) in the States and the politics (Political Score, PS-Democrat/Republican Ratio) as well as the restrictions (Restriction Score, RS) of that State

Large differences were detected between the states regarding the degree of COVID restrictions and the associated epidemiological parameters. It directly opens the interest for further discovery to find the human factors behind these differences when the targeted virus was essentially the same in every states. “Save the vulnerable” (i.e. elderly) and those having chronical health conditions is the primary motivation of all restrictions and the significant personal offerings of the younger generations. However there is no statistically detectable influence of elderly, neither on the states politics nor on the degree of restrictions (Figure 6). Ethnicity is a well-known indicator of human behavior. ‘White’ is the largest ethnical group in the USA (~ 60 % of all), therefore the simple white/non-white ratio was used to see the possible effects of ethnicity on the scores used in this study. The proportion of ‘whites’ in the states reversely correlated with the democrat orientation and the number of restrictions in the states: More ‘whites’ indicates less democrats and less restrictions (Figure 7). Religion is also a major determinant of human behavior. Protestants is the largest religious group in the USA (~ 43% of all), therefore the simple protestant/non-protestant ratio was used. The proportion of Protestants in the states reversely correlated with the democratic orientation and the number of restrictions in the states: More Protestants indicates less democrats and less restrictions (Figure 8). The size of the Jewish population showed strong positive correlation with the democrat orientation and strict restrictions in the states. More Jewish citizens indicated more democrats and more restrictions in the State in question (Figure 9). Increasing Medicaid spending [Federal money] is associated with the more democrat orientation as well as with more restrictions. This tendency is less detectable regarding the total health spending of the respective states (Figure 10).

### The big picture

**Figure 11:**
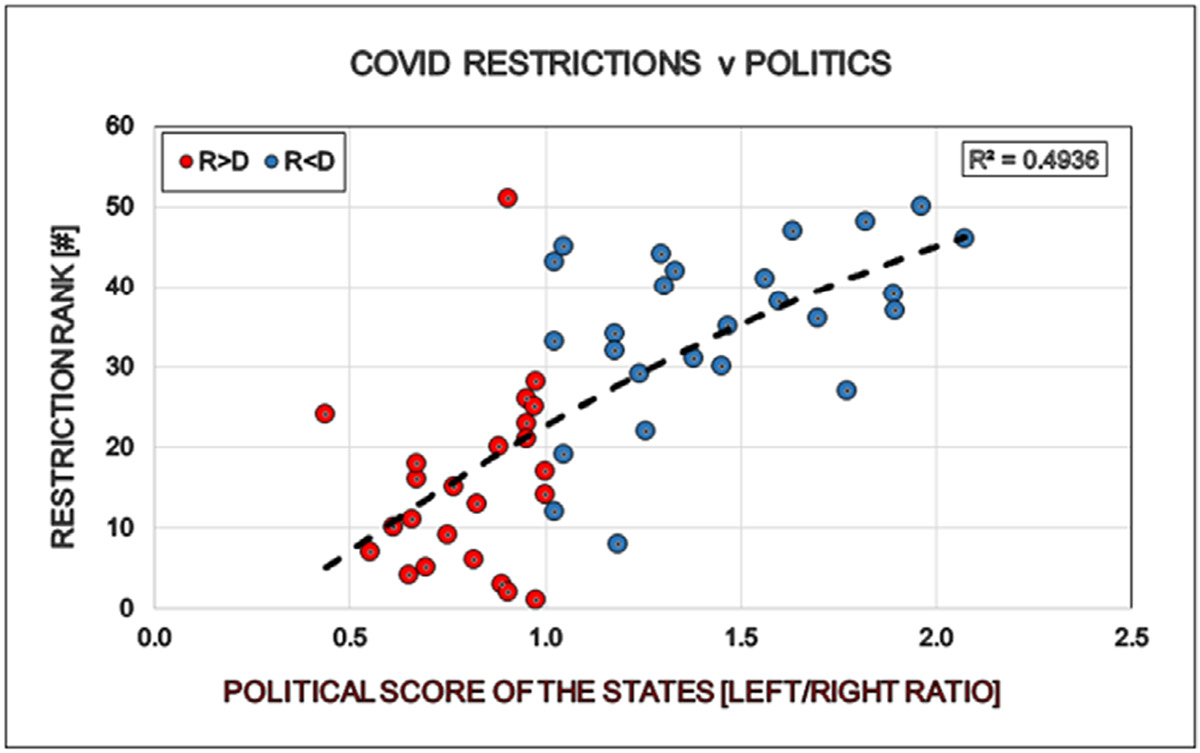
Correlation between politics (Political Score, PS: Democrat/Republican Ratio) and restrictions (Restriction Rank/Score, RS) of the States

There is a strong positive correlation between the political bias of the States and the grade of restrictions in the respective states. More deviation toward left in a State results in more restrictions in that State (Figure 11).

## Discussion and Conclusion

It was possible to observe, that significant differences exist between ‘red’ and ‘blue’ states regarding the involvement in the Covid epidemics. Democrat states were much harder effected by the virus, than republican States. We minted the term “Politi-Covid” in our earlier analyses [4]. The Covid epidemic brutally interfered with the presidential election and resulted in very serious political complications [8]. The virus continued its political carrier end resulted in continued serious restrictions of our freedom and access to our normal work and regular social contacts. The theory behind the restrictions was, that we all accepted, that we have to save our elderly and other sick persons from COVID infection and the supposedly associated untimely death. The introduction of qualitative statistical methods to the objective and non-partisan studies on the real results of the restrictions and the driving forces behind revealed that practically all of our restrictions were politically decided and enforced by the left oriented citizens. These restrictions (personal offerings by the citizens) didn’t result in any detectable reduction of the Covid mortality. The complete absence of any reduction of Covid deaths was observed first by other investigators and we could confirm it: Covid restrictions had no statistically detectable epidemiologic advantage for the “participating” Americans [6]. A remarkable observation is the increased (SIC!) of COVID lethality in strongly restrictive and blue states. When the number of COVID infected (test positive) positive persons is reduced and is reasonably expected that the mortality and lethality will follow (Figure 3). It didn’t happen: a) Mortality [deaths/100K persons, healthy and sick] remained unchanged (Figure 4); b) lethality [deaths/100 infected and sick persons] increased instead (Figure 5). This result doesn’t make any sense except if we assume, that determination of the Underlying Cause of Death (UCOD) was not exclusively based on positive laboratory tests (clinical, laboratory evidence) but it was diagnosed even in the absence of test-confirmation. The observation of “hearsay” COVID deaths continued even when the “supply” of true COVID infected deaths was reduced (by the restrictions?). The result is the virtual increase of lethality, a paradoxical effect that was possible only in some blue states. The logical interpretation of the disappointing stagnation of COVID deaths may be that the strongly criticized “hearsay based diagnoses of COVID deaths” remained in use for a long time [7].

- Hearsay based diagnosis is when the determination of the UCOD is based on the assumption that the person was exposed to the virus, but it was never confirmed by specific laboratory test. Hearsay is the information received from other people that one cannot adequately substantiate; rumor. The report of another person's words by a witness, which is usually disallowed as evidence in a court of law.

Reduction of total Covid cases will be positively interpreted by the supporters of further restrictions, however the value of this result is strongly questionable in the complete absence of reduction of the Covid associated death-rate. It is of course valuable to see the same driving forces behind restrictions and politics, namely the preferential influence of the a) Judeo-Catholic (non-protestant) population, b) the non-white ethnical groups as well as the c) health-economic interest. The strong influence of the above factor on the people’s behavior is not new and exhaustively discussed by others. The above results suggest that the influence of our subjective mind on our most important decision-making processes (here the defense strategy against a virus threat) is large and polarizing. The technical and economic development in the USA made it possible the early end effective use of laboratory tests for correct COVID diagnostic, the sophisticated computer technology made it possible to turn raw data from the “field” into usable high-quality epidemiologic information followed by qualified decision making. It is depressing to see that the world’s largest democracy and richest country was/is ignorant to utilize its own resources – in some aspects and some places and returned to the niveau of less fortunate countries (like utilizing “hearsay diagnostic” as country doctors used to do for a century ago). The general suggestion (conclusion) of our statistical study is that the main difficulty to handle the COVID epidemic was not the virus itself, but its complex, syndemic nature that was supposedly too difficult to manage by the appointed decisionmakers of the time [5]. There remains of course-a big question to answer: How and when will this great American Federation recover from the consequences of the virus itself and the not always fortunate human interferences. COVID-19 virus caused “A Once-in-a-Century Pandemic?” as Bill Gates called it in the respectable N Engl J Med. [9]. Others expressed worry over that we are dealing with “A once in a century evidence fiasco” instead [10]. Today we can exclude the first possibility, but the second is still possible. Ioannidis [leading expert of biostatistics at Stanford] was right already in 2020: “We are [were] making decisions without reliable data.”
